# Development and Delphi validation of WONCA-aligned competency-based learning outcomes for undergraduate family medicine internship

**DOI:** 10.3389/fmed.2026.1899210

**Published:** 2026-07-15

**Authors:** Mariana Bento, Joana Monteiro, Paulo Santos

**Affiliations:** 1 Faculty of Medicine, University of Porto, Alameda Prof. Hernâni Monteiro, Porto, Portugal; 2 MEDCIDS, Faculty of Medicine, University of Porto, Alameda Prof. Hernâni Monteiro, Porto, Portugal; 3 RISE-Health, Faculty of Medicine, University of Porto, Alameda Prof. Hernâni Monteiro, Porto, Portugal

**Keywords:** clinical competency, competency-based education, Delphi method, family practice, undergraduate medical education

## Abstract

**Introduction:**

Competency-Based Medical Education (CBME) promotes the integration of knowledge, skills, and attitudes through an outcomes-oriented approach. Family Medicine offers an ideal context for its implementation; however, undergraduate training remains heterogeneous and insufficiently aligned with the World Organisation of Family Doctors (WONCA) competency framework. In particular, assessment often remains focused on task completion rather than observable performance in authentic clinical settings. This study aimed to develop and validate WONCA-aligned competency-based learning outcomes for undergraduate Family Medicine to support more explicit and competency-oriented clinical training.

**Methods:**

A structured multi-phase study combining expert-informed item generation and Delphi-based consensus validation was conducted. In the initial phase, Family Medicine physicians with experience in medical education contributed proposed learning outcomes through an open-ended questionnaire. These were synthesised into preliminary outcomes aligned with the WONCA competencies. A two-round modified Delphi process was then used to evaluate each outcome using a five-point Likert scale. Content validity was assessed using the item-level content validity index (I-CVI), with consensus defined as ≥0.80.

**Results:**

Twenty-two preliminary learning outcomes were generated. In the first Delphi round (*n* = 109), 20 outcomes achieved consensus (I-CVI ≥ 0.80). Two outcomes required reformulation and were re-evaluated in a second round (*n* = 103), after which both reached consensus. The final set comprised 22 validated competency-based learning outcomes aligned with the WONCA framework.

**Conclusion:**

This study provides a Delphi-validated operationalisation of the WONCA competency framework into observable and developmentally appropriate learning outcomes for undergraduate Family Medicine. These outcomes offer a structured foundation for competency-oriented teaching and assessment, and may inform the future development of portfolio-based approaches in clinical education.

## 1 Introduction

Over the last decades, rapid transformations in society, technology, and healthcare systems have reshaped expectations for medical education and the competencies future physicians must acquire. Medical schools play a central role in preparing graduates with the necessary knowledge, skills, attitudes, ethical awareness, and humanistic perspective required for contemporary clinical practice (1–4).

Traditionally, medical education has been tutor-centred, emphasising knowledge acquisition through lectures, clinical observation, and structured assessments (2, 5, 6). While these approaches are effective for assessing knowledge, particularly in the early years, they have been criticised for focusing on curricular completion rather than the development of meaningful professional competence applicable to real-world practice (6). This concern has been reinforced by increasing demands for high-quality care and accountability within healthcare systems (7). Earlier calls for reform, such as the Edinburgh Declaration, emphasised the importance of aligning curricula with professional competence, active learning, and socially responsive medical training (8). Together, these developments highlight the need to reconsider how medical students are trained and assessed.

The shift toward competency-based medical education (CBME), consolidated in the early 21st century, reflects this need by prioritising outcomes that integrate knowledge, skills, and attitudes in authentic clinical contexts (5–7, 9, 10). In CBME, competencies define the broad domains of professional practice, while learning outcomes specify the observable behaviours that demonstrate these competencies (4, 11). This approach emphasises active learner engagement, shared responsibility between students and tutors, and the use of feedback to guide professional development (5, 6).

Miller’s pyramid of clinical competence further supports this framework by distinguishing progressive levels of competence, from knowledge acquisition to performance in practice (12–15). In this context, assessment methods must move beyond theoretical knowledge to include performance evaluation in real clinical settings. Workplace-based assessment methods, including direct observation, enable such evaluation, while structured tools such as portfolios can support longitudinal learning, reflection, and integration of feedback (6, 16–18).

Family Medicine offers a particularly suitable context for implementing CBME principles during undergraduate education. It provides exposure to person-centred, continuous, comprehensive, and community-oriented care in real clinical environments (19, 20). Undergraduate training in Family Medicine contributes to a better understanding of healthcare systems, continuity of care, and preventive approaches, while also influencing career choice and professional identity (21–23). European recommendations emphasise the importance of incorporating the core characteristics of Family Medicine into undergraduate curricula and promoting meaningful clinical experiences (24). However, translating these principles into structured learning and assessment strategies remains challenging (9, 13).

The WONCA European Definition of General Practice/Family Medicine provides an internationally recognised framework describing the competencies and attributes required for the discipline (25). It includes six core competency domains—primary care management, person-centred care, specific problem-solving skills, a comprehensive approach, community orientation, and holistic care—supported by additional characteristics and foundational competencies (25). The WONCA competency tree illustrates the breadth of expertise expected of Family Medicine. Its leaves represent the defining characteristics of the discipline: holistic and longitudinal care, first-contact with open access for all health problems, care coordination and advocacy, health promotion and wellbeing, management of acute and chronic health problems, care in early and undifferentiated stages of illness, decision-making based on incidence and prevalence, community health responsibility, patient empowerment, care centred on the patient and context and the doctor-patient relationship (25). These characteristics are supported by the central professional tasks of clinical care, communication, and practice management, and are rooted in science, context, and professional attitude (25). This structure reinforces the idea that undergraduate Family Medicine training should expose students to the full scope of the discipline while translating these expectations into learning outcomes appropriate to their level of autonomy. Although widely accepted, this framework is primarily conceptual and requires operationalisation to be effectively applied in undergraduate clinical training.

For undergraduate education, these competencies must be translated into explicit, observable, and assessable learning outcomes that are appropriate to students’ level of autonomy and feasible within short clinical placements. Defining such outcomes is essential to clarify expectations for student performance and to support competency-based teaching and assessment during clinical internships.

In Portugal, medical education is organised as a six-year integrated master’s degree. Early years focus on biomedical sciences, followed by progressive clinical training across specialities. At the Faculty of Medicine of the University of Porto (FMUP), Family Medicine is introduced in the fourth year. Still, the sixth-year internship represents the main opportunity for immersive clinical experience. This four-week internship takes place in primary care settings under the supervision of Family Medicine physicians serving as clinical tutors. However, variability exists in tutor training and assessment practices. Student evaluation typically emphasises attendance, predefined activities, written assignments, and tutor judgement, with less structured observation of competency development in real clinical encounters.

As in many European contexts, this results in an internship that remains relatively unstandardized and often oriented toward knowledge acquisition rather than the progressive demonstration of integrated competencies aligned with the WONCA framework (22). This gap highlights the need for clearer, competency-based learning expectations that can guide both teaching and assessment in Family Medicine.

Despite the relevance of the WONCA framework, there is limited evidence on how its competencies can be systematically translated into validated learning outcomes for undergraduate education, using a consensus approach to clarify which topics are most characteristic of the speciality and to serve as assessment markers.

Addressing this gap is essential to bridge the disconnect between competency frameworks and their practical application in clinical training. Developing such outcomes can provide a structured and internationally grounded reference for clinical education, supporting more explicit, observable, and competency-oriented learning during Family Medicine internships. These outcomes may also inform the future development of structured approaches to assessment and learning, including portfolio-based models that integrate clinical experience, feedback, and reflection (26).

This study aimed to develop and validate, through expert consensus, a set of WONCA-aligned competency-based learning outcomes for the sixth-year Family Medicine internship, thereby providing a structured foundation for competency-oriented undergraduate training.

## 2 Materials and methods

### 2.1 Study design

We conducted a structured multi-phase study combining expert-informed item generation and Delphi-based consensus validation. An initial item-generation phase was used to develop preliminary competency-based learning outcomes aligned with the WONCA framework, followed by a validation phase using a modified Delphi approach to assess each learning outcome individually (27).

Elements of the item-generation phase are described in accordance with established reporting practices to ensure transparency and reproducibility (28). In contrast, the Delphi validation phase follows the DELPHISTAR guidelines for Delphi studies (29).

Data from the first exploratory questionnaire were collected in January 2025. The first Delphi round was conducted between April and June 2025, followed by a second round in July 2025.

The questionnaires were administered in Portuguese. For publication purposes, the research team translated the final validated learning outcomes into English and reviewed them for conceptual equivalence with the original Portuguese formulations.

### 2.2 Participants

Participants in the item-generation phase were selected using purposive sampling. Eligibility criteria included being a Family Medicine physician with prior or current involvement in undergraduate or postgraduate medical education within the preceding 12 months. Teaching involvement could include clinical tutoring, supervision of undergraduate or postgraduate trainees, participation in teaching activities, or involvement in assessment processes. In Portugal, the Family Medicine career progresses through formally defined grades, specialist, consultant, and senior consultant, based on years of practice and accumulated merit. These criteria were defined to ensure that participants had practical familiarity with Family Medicine training, clinical supervision, and the educational context in which the proposed learning outcomes would be implemented. At FMUP, the sixth-year Family Medicine clerkship lasts 4 weeks and takes place in primary care settings under the supervision of Family Medicine clinical tutors. The decision to include physicians involved in postgraduate training alongside those involved in undergraduate education was deliberate, as in the Portuguese primary care setting, Family Medicine physicians often supervise both undergraduate students and postgraduate residents, and their clinical supervision experience was considered relevant to the evaluation of undergraduate learning outcomes. This broader eligibility criterion was intended to ensure a sufficiently large and experienced expert panel while maintaining the focus on outcomes appropriate for sixth-year medical students. Participants were recruited via electronic invitations distributed through professional and academic networks. Participation was voluntary, and the final sample size reflected the number of eligible respondents who completed the questionnaire within the study period.

For the modified Delphi rounds, Family Medicine physicians were invited through the same dissemination strategy. Eligibility for inclusion in the analysis required current or recent involvement in undergraduate or postgraduate medical education, in line with the study objective of validating learning outcomes for undergraduate training. Although responses were received from a broader group, only those meeting the predefined eligibility criteria were included in the analysis. Responses from individuals without documented teaching experience were excluded.

### 2.3 Data collection procedure

The study was organised into three sequential phases.

Phase 1 – Item-generation phase:

First, a qualitative exploratory phase was conducted, during which a semi-structured online questionnaire was distributed to a purposive sample of Family Medicine physicians with previous or current involvement in undergraduate or postgraduate teaching. The objective was to identify learning outcomes that could demonstrate the acquisition of competencies described in the WONCA definition. In the item-generation questionnaire, participants were presented with components of the WONCA competency tree, including 18 open-ended items to address the 12 WONCA-specific competencies, the 3 central trunk competencies, and the 3 foundational domains. For each WONCA competency or domain, participants were asked to provide one example of what a sixth-year medical student could perform during the Family Medicine internship to demonstrate acquisition of that competency. This structure allowed participants to generate concrete undergraduate-level tasks while ensuring that all elements of the WONCA definition were addressed. Because the study’s purpose was to operationalise the WONCA framework rather than to develop a new competency model, participants were not asked to propose additional competency domains outside the WONCA structure. A brief section on sociodemographic and professional background was also included.

Phase 2 – Development of preliminary learning outcomes:

In the second phase, the three researchers involved in the study reviewed and synthesised the responses from the item-generation phase. The research team comprised three authors with complementary perspectives: a sixth-year medical student enrolled in the Integrated Master’s Degree in Medicine at the Faculty of Medicine of the University of Porto, a Family Medicine specialist and invited lecturer at the MEDCIDS Department of the Faculty of Medicine of the University of Porto and a Family Medicine specialist and Professor at the Faculty of Medicine of the University of Porto. This combination brought together the perspective of an undergraduate learner, clinical experience in Family Medicine, and academic expertise in medical education. The aim was not to create new competency domains, but to translate the existing WONCA competencies into appropriate and observable learning outcomes for undergraduate Family Medicine training. Responses for each WONCA competency domain were grouped and read independently by each researcher, who then identified the most recurrent and conceptually relevant proposed activities. Items were retained if they were clearly described as observable, contextually appropriate for a short primary care placement, and directly aligned with the corresponding WONCA competency. Generic items, duplicated across domains, or not feasibly observable during an undergraduate clerkship, were excluded. Following discussions amongst the research team, a preliminary set of observable learning outcomes was generated. In some domains, multiple learning outcomes were generated to reflect different perspectives or to provide alternative formulations for subsequent validation. This decision was taken in domains where the inherent complexity of the competency could not be adequately captured through a single activity, thus justifying its division into complementary competencies. Discrepancies between researchers were resolved through iterative discussion until consensus was reached.

Phase 3 – Quantitative phase:

Finally, a quantitative validation phase was conducted using a modified Delphi technique. Two sequential online questionnaire rounds were distributed to the same panel of Family Medicine physicians to validate each learning outcome individually through structured expert consensus.

The research team disseminated invitations electronically through professional and academic networks. As is commonly observed in Delphi studies, the number of eligible respondents varied slightly across rounds due to non-response and changes in eligibility criteria; only participants who met the predefined inclusion criteria were included in the analysis for each round.

The first Delphi round consisted of a structured online questionnaire based on the preliminary learning outcomes developed in the previous phase. Participants were asked to rate their level of agreement with each statement using a five-point Likert scale, ranging from 1 (“strongly disagree”) to 5 (“strongly agree”). The questionnaire also collected sociodemographic and professional information and included an open-ended section for additional comments or suggestions. While this section was not intended for participants to propose new learning outcomes, as item generation had been completed in the initial phase, the research team reviewed the free-text comments to guide the reformulation of specific learning outcomes that did not reach the predefined consensus threshold. The research team reformulated learning outcomes that did not reach the predefined consensus threshold. Participants were informed via email that consensus had not been achieved for these items and were invited to re-evaluate the revised items in a second Delphi round using a new online questionnaire. Professional status was not reassessed in the second Delphi round because it was distributed to the same population as in the first round and was limited to the re-evaluation of the two reformulated learning outcomes.

### 2.4 Data analysis

Open-ended questionnaire responses were analysed using a directed content analysis approach, with the WONCA competency domains used as the initial analytical framework (30). During item synthesis and refinement, the team was aware that its educational and professional backgrounds could influence interpretation. To support a systematic, transparent process, responses were first organised by the corresponding WONCA competency or domain. Within each domain, the three researchers reviewed the responses, identified recurrent and conceptually similar ideas, and synthesised them into concise preliminary learning outcomes describing activities that sixth-year medical students could observe, perform, discuss, or reflect on during the Family Medicine internship. The research team prioritised clarity, relevance to undergraduate clinical teaching, suitability within a short primary care placement, alignment with the corresponding WONCA competency, and appropriateness to the expected level of autonomy of sixth-year students. Individual suggestions were integrated, reformulated, or not retained as separate items when they were duplicative, insufficiently observable or discussable within the clerkship, substantially overlapping with another retained item, or more appropriate for postgraduate rather than undergraduate training.

Descriptive statistics were used to analyse quantitative data from the Delphi rounds. Content validity was assessed using the item-level content validity index (I-CVI), calculated as the proportion of experts rating each learning outcome as 4 or 5 on a 5-point Likert scale, in accordance with established CVI methodology (31). Ratings of 4 or 5 were considered indicative of agreement with item appropriateness, whereas ratings of 1–3 were classified as non-agreement. Therefore, the I-CVI was interpreted as an item-level agreement proportion reflecting content validity. The Statistical Package for the Social Sciences (SPSS) version 30.0.0.0 was used to compute agreement proportions for each learning outcome. A predefined threshold of ≥0.80 was used to indicate acceptable content validity and consensus, consistent with commonly accepted standards for item-level content validity (32).

When consensus was not achieved, the researchers reformulated the statements based on insights from the initial item-generation phase. They re-evaluated them in a new Likert-scale validation round using the same procedures until the minimum agreement threshold was reached.

A secondary analysis was also performed, including only participants who reported current involvement in undergraduate medical education, regardless of concurrent participation in postgraduate training, to explore potential differences in the type of involvement.

### 2.5 Ethical issues

This study was submitted and approved by the Ethics and Research Committee of the Faculty of Medicine of the University of Porto (protocol number 282/CEFMUP/2024).

The study followed all the principles of the Helsinki Declaration and Oviedo Convention for conducting research involving human beings. Informed consent was obtained electronically from all participants, who were fully briefed on the study’s objectives and methodology and assured of the confidentiality and anonymity of their participation. Data were collected via online questionnaires in Google Forms®. Participation was voluntary, and respondents could withdraw at any time without any consequence.

## 3 Results

A total of 17 Family Medicine physicians from multiple regions participated in the initial item-generation phase. The majority practised in the North of Portugal (*n* = 9), with the remaining participants distributed across the Centre (*n* = 4) and South (*n* = 4).

In the first modified Delphi round, 117 responses were received, of which 109 met the predefined eligibility criteria and were included in the analysis. In the second round, 113 responses were received, of which 103 were eligible and included in the final analysis. The sociodemographic and professional characteristics of participants across the three study stages are summarised in Table 1. The participants’ professional profiles further strengthened the panel’s expertise. In the item-generation phase, 12 of 17 participants had more than 10 years of experience in Primary Health Care, and 12 were Family Medicine consultants or senior consultant physicians. In the first round, 78 of 109 participants had more than 10 years of experience, with 55 Family Medicine specialists and 54 Family Medicine consultants or senior consultant physicians.

**Table 1:** Sociodemographic and professional characteristics of participants across study phases.

Sociodemographic and professional characteristics	Category	Subcategory	Item-generation phase *n* = 17	Delphi round 1 *n* = 109	Delphi round 2 *n* = 103
Sex	Female		8 (47.1)	71 (65.1)	68 (66.0)
	Male		9 (52.9)	38 (34.9)	35 (34.0)
Age, years	Mean [range]		46 [34–71]	42 [32–70]	41 [32–68]
Years of experience in Primary Health Care	≤10 years		5 (29.4)	31 (28.4)	–
	>10 years		12 (70.6)	78 (71.6)	–
Current involvement in undergraduate or postgraduate medical education	Yes	Total (undergraduate + postgraduate)	14 (82.4)	109 (100)	103 (100)
		Specifically, in undergraduate medical education	13 (76.5)	78 (71.6)	62 (60.2)
	No		3 (17.6)	–	–

Following the item-generation phase, the research team developed 22 preliminary competency-based learning outcomes aligned with the WONCA European Definition of General Practice/Family Medicine. These outcomes were designed to translate the WONCA framework into observable expectations for sixth-year medical students during the Family Medicine internship.

Based on the open-ended responses addressing the 18 WONCA competencies, a total of 22 learning outcomes were generated for subsequent validation, as presented in Table 2. In some domains, more than one learning outcome was retained, reflecting different aspects of the same competency and explaining the total number exceeding the number of original competency domains.

**Table 2:** Preliminary learning outcomes aligned with WONCA competencies and item-level content validity indices (I-CVI) in the total sample (*n* = 109) and in the undergraduate teaching subsample (*n* = 78).

Core competencies from the WONCA definition	Specific competencies	Corresponding learning outcomes	Doctors involved in medical education I-CVI (%) T = 109	Doctors involved in undergraduate education I-CVI (%) T = 78
Person-centred care	Doctor–patient relationship	1. In the clinical interview with the patient, the student adopts a person-centred approach to medicine	94.5	93.6
	Centred on the patient and context	2. The student analyses personal, family, social and community resources, ensuring that the plan is appropriate to the patient’s circumstances.	89.9	88.5
	Promotes patient empowerment	3. The student promotes health literacy and checks the patient’s understanding of how to manage their problems.	90.8	89.7
		4. During the consultation, the student takes a proactive approach, involving the patient in the decision-making process and ensuring that they understand the treatment plan.	90.8	89.7
	Longitudinal Continuity	5. The student places the current episode of care seeking within the context of the patient’s health history.	94.5	93.6
Community orientation	Responsible for the health of the community	6. The student guides individual care, taking into account the health priorities of the community in which they live.	71.6	70.5
Specific problem-solving skills	Decision-making based on incidence and prevalence	7. The student is able to identify the most prevalent health problems in the community and adapt the diagnostic and therapeutic approach to the local context.	87.2	85.9
		8. The student orders differential diagnoses based on clinical and epidemiological assessment and the patient’s context.	98.2	98.7
	Early undifferentiated stages	9. Students analyse clinical cases, developing open and contextualised clinical reasoning capable of integrating diagnostic uncertainty.	94.5	92.3
Comprehensive approach	Acute and chronic health problems	10. The student conducts acute illness consultations and chronic illness monitoring consultations under the direct supervision of the tutor.	80.7	85.9
	Promotes health and well-being	11. The student integrates preventive measures and screening appropriate to the individual’s context, at different levels of prevention.	91.7	91.0
Primary care management	Care coordination and advocacy	12. The student recognises the role of the family doctor in the healthcare system.	93.6	91.0
		13. The student assesses the need for referral to other levels of care from the perspective of integrated care.	93.6	94.9
	First-contact, open access, all health problems	14. The student identifies the reasons for seeking medical and non-medical health services.	90.8	89.7
		15. The student understands the journey of individuals within the healthcare system.	83.5	83.3
Holistic modelling	– Physical, psychological, social, cultural, and existential dimensions	16. Students explore patients’ beliefs, values, and psychosocial context, integrating cultural, religious, and social aspects into the assessment and treatment plan.	84.4	83.3
Central trunk competencies				
Clinical tasks		17. The student collects the clinical history in a structured manner, performs a physical examination focused on the patient’s problem, and proposes an appropriate care plan under the supervision of the tutor.	92.7	92.3
Communication with patients		18. The student uses appropriate verbal and non-verbal communication tools, promoting empathetic communication.	97.2	96.2
Management of the practice		19. The student is familiar with the model for organising a doctor’s weekly schedule.	76.1	73.1
Foundational domains (roots)				
Attitude		20. The student demonstrates diligence, punctuality and professionalism, reflects on the impact of their beliefs on medical practice and identifies gaps in knowledge to improve their performance.	96.3	94.9
Science		21. The student analyses the risks and benefits of tests and treatments and proposes strategies based on the best available medical evidence.	91.7	91.0
Context		22. The student understands the influence of the organisational context on medical practice.	84.4	84.6

Table 2 presents the results of the first Delphi round, including the primary analysis of all participants who met the inclusion criteria (*n* = 109) and a secondary analysis of those involved in undergraduate teaching (*n* = 78). In the primary sample, 20 of 22 learning outcomes achieved an I-CVI ≥ 80%, indicating high consensus. Items 6 and 19, corresponding to the domains “Responsible for the health of the community” and “Management of the practice,” did not reach the predefined threshold.

The secondary analysis, restricted to participants involved in undergraduate medical education, yielded consistent results, with the same two learning outcomes remaining below the consensus threshold. These findings indicated that items 6 and 19 required revisions, as the lack of consensus persisted even amongst respondents with more direct involvement in undergraduate teaching.

The research team subsequently reformulated learning outcomes 6 and 19 to improve clarity, alignment with teaching practice, and representativeness of the corresponding WONCA competency domains, as presented in Table 3. The revised items were incorporated into a new structured questionnaire and redistributed to the same target population for the second Delphi round.

**Table 3:** Revised learning outcomes 6 and 19 and item-level content validity indices (I-CVI) in the total sample (*n* = 103) and in the undergraduate teaching subsample (*n* = 62).

Core competencies from the WONCA tree	Specific competencies	Corresponding learning outcomes	Doctors involved in medical education I-CVI (%) T = 103	Doctors involved in undergraduate education I-CVI (%) T = 62
Community orientation	Responsible for the health of the community	6. The student recognises the health resources available in the community.	81.6	79.0
Central trunk competencies				
Management of the practice		19. The student prepares a structured record of a consultation period in which they identify the organisational strategies of the clinical activity observed.	94.2	91.9

Table 3 presents the results of the second Delphi round for the reformulated learning outcomes, including the primary analysis (*n* = 103) and a secondary analysis restricted to participants involved in undergraduate teaching (*n* = 62). In the primary analysis, both revised items achieved consensus (I-CVI ≥ 80%), meeting the predefined validation criteria.

In the secondary analysis, one learning outcome reached a slightly lower level of agreement (79%). However, as predefined in the study protocol, validation decisions were based on the primary analysis.

The final set of validated competency-based learning outcomes is provided as Supplementary material.

## 4 Discussion

The present study developed and validated 22 competency-based learning outcomes aligned with the WONCA European Definition of General Practice/Family Medicine (33). These outcomes should be interpreted as a contextual operationalisation of an established international competency framework, rather than as the proposal of a new model.

Unlike previous work, this study provides a Delphi-validated, context-specific operationalisation of the WONCA competency framework tailored to undergraduate training. To our knowledge, this is one of the first studies to translate these domains into observable and developmentally appropriate learning outcomes for Family Medicine, addressing a recognised gap between competency frameworks and their practical application in clinical education.

The engagement of a large panel of Family Medicine physicians, together with the substantial levels of agreement observed across Delphi rounds, supports the perceived relevance and appropriateness of translating WONCA competencies into structured undergraduate learning outcomes. However, expert consensus and content validity should not be interpreted as evidence of educational effectiveness. The I-CVI values should therefore be interpreted as evidence that the proposed outcomes reached predefined thresholds of expert agreement, supporting content validity, rather than as evidence that they are feasible to implement, reliably assessable, or educationally effective in clinical placements. This study does not establish whether implementation of these learning outcomes improves student performance, feedback quality, assessment consistency, or learning during clinical placements. In addition, although the outcomes were designed to be observable or discussable within clinical encounters, participants were not asked to rate observability or assessability as separate dimensions. The extent to which each learning outcome can be reliably assessed through direct observation, therefore, remains to be established. Most learning outcomes reached consensus in the first round, supporting the adequacy of the initial framework. The predefined I-CVI threshold of ≥0.80, consistent with widely accepted recommendations in content validity studies, reflects a deliberate balance between ensuring strong expert agreement and avoiding excessive exclusion of potentially relevant learning outcomes. This approach supports the robustness of the final set while preserving its practical applicability in educational contexts. The two outcomes requiring reformulation, related to community orientation and practice management, reflect areas that are inherently more difficult to operationalise at the undergraduate level, as they involve broader organisational and population-level dimensions of practice. Importantly, no WONCA competency was considered irrelevant for undergraduate exposure. All 18 domains were retained and operationalised into at least one learning outcome, reflecting the panel’s view that even foundational-level competencies are appropriate to include in undergraduate level.

The refinement of these outcomes clarified expected levels of student performance. Although one reformulated outcome showed slightly lower agreement in the subgroup of participants involved in undergraduate teaching, validation decisions were based on the predefined criteria applied to the full expert panel, ensuring consistency with the study protocol. The revised formulations emphasised observable learning outcomes aligned with students’ levels of autonomy and the realities of short clinical placements. This process reinforces the importance of iterative development when translating broad competency frameworks into practical educational tools.

By providing a structured set of learning outcomes, this work supports a shift from teaching about competencies to teaching and assessing through competencies, thereby promoting more authentic preparation for clinical practice (3, 9). Each outcome operationalises a specific WONCA domain, strengthening alignment with internationally recognised expectations for undergraduate Family Medicine education (21).

Although the WONCA framework offers a comprehensive conceptual description of the discipline, its educational value depends on its translation into activities that can be realistically experienced during clinical placements. Given the limited duration and variability of undergraduate internships in primary care, it is not feasible for students to be exposed to the full range of clinical situations. Defining a set of representative learning outcomes allows meaningful assessment within real-world constraints while maintaining alignment with the breadth of the discipline (34).

Previous European recommendations have highlighted the need for undergraduate curricula grounded in the core characteristics of Family Medicine (24), but do not provide validated learning outcomes tailored to short clinical placements. Similarly, prior work has focused predominantly on postgraduate training or broader programmatic frameworks (35, 36). This study addresses this gap by providing a validated set of outcomes specifically designed for undergraduate Family Medicine internships.

These learning outcomes also inform structured approaches to teaching and assessment in undergraduate training. Portfolio-based models offer a promising framework in this context, in which each learning outcome could be linked to one or more documented clinical encounters and students could be asked to document the acquisition of WONCA-aligned learning outcomes, engage in self-reflection, and receive structured tutor feedback, enabling the portfolio to serve as both a learning and an assessment tool (18, 26). Students could also record the clinical context and identify the WONCA-aligned outcome addressed. For example, clinical reasoning outcomes could be assessed through case-based discussion, communication outcomes through direct observation of consultations, and practice-management outcomes through a structured record of an observed consultation. Workplace-based assessment approaches, including direct observation, mini-clinical evaluation exercises, and case-based discussion, may also be supported by these outcomes, as they clarify what students are expected to observe, perform, discuss, or reflect on during the internship (16). Their educational value depends on active engagement of both students and tutors, particularly through feedback processes that support self-regulated learning and professional development (37, 38).

Although developed within the Portuguese context, alignment with the WONCA framework supports the potential transferability of these outcomes to other settings. WONCA provides an internationally recognised disciplinary reference for General Practice/Family Medicine, and its competency framework offers a shared language for describing the speciality’s core features (25). Therefore, the learning outcomes developed in this study may be relevant to other medical schools seeking to operationalise Family Medicine competencies in undergraduate clinical training and may help to reduce ambiguity between intended learning objectives and observed clinical performance. However, direct adoption in other countries or institutions should not be assumed. Differences in curriculum structure, duration of Family Medicine placements, student autonomy, primary care organisation, tutor preparation, and assessment culture may require contextual adaptation and local validation before implementation. In this sense, the proposed outcomes should be viewed as a transferable framework rather than a universally prescriptive instrument.

Beyond Family Medicine, this approach may be adaptable to other clinical disciplines. Competency-based models have been shown to support integration across the continuum of medical education and may contribute to greater coherence between learning objectives and assessment strategies (39, 40). However, effective implementation depends on clinical tutors’ preparedness to support competency-based learning. Teaching, supervision and feedback require specific pedagogical competencies that extend beyond clinical expertise, highlighting the importance of faculty development (3, 41).

The validated learning outcomes may also serve as a foundation for future development of Entrustable Professional Activities (EPAs), which integrate multiple competencies into units of professional practice (42, 43). Although originally developed for postgraduate training, EPAs are increasingly applied in undergraduate education to clarify expectations and support progressive autonomy (43–46). For example, the learning outcome requiring the student to collect a structured clinical history, perform a focused physical examination, and propose a supervised care plan could contribute to a future undergraduate EPA, such as “conducting a supervised Family Medicine consultation for a common acute or chronic problem.,” which would integrate clinical reasoning, communication, patient-centred care, and management planning. Mapping learning outcomes onto EPA structures in this way may help bridge the current gap between competency frameworks and actionable assessment tools. However, the present study did not define entrustment levels or entrustment decisions, which would require further implementation and validation work. Future research should focus on implementation studies evaluating how these learning outcomes function in real clinical teaching settings. Such studies should involve students, clinical tutors, and curriculum leaders, and should examine feasibility, usability, tutor workload, quality of feedback, assessment consistency, and perceived impact on learning. Further work should also determine which learning outcomes require direct observation, which may be better assessed through case-based discussion or portfolio reflection, and how many documented encounters or observations are needed to support credible judgements within the limited duration of the clerkship.

A key strength of this study lies in the size, geographic breadth, and educational backgrounds of the expert panel, which enhance the credibility and content validity of the outcomes. The use of professional and academic networks was appropriate for reaching Family Medicine physicians with direct experience in teaching, supervision, and assessment, and therefore familiar with both the competencies required in Family Medicine and the practical constraints of undergraduate clinical placements. The use of a structured Delphi process allowed systematic consensus-building amongst experienced clinicians (47). However, participation was voluntary and may have introduced self-selection bias, potentially favouring respondents with a stronger interest in medical education and may also have contributed to the high levels of consensus observed, as participants recruited through shared professional and academic networks may hold more homogeneous views on Family Medicine training than the broader population of clinical supervisors. In addition, the panel was not deliberately stratified according to academic rank, institutional role, or level of educational leadership. Therefore, some perspectives within the educational hierarchy may have been unevenly represented. Including physicians involved in postgraduate education may have introduced perspectives influenced by expectations beyond undergraduate training. However, this overlap reflects the Portuguese primary care teaching context, in which the same Family Medicine physicians often supervise both undergraduate students and postgraduate residents. Moreover, the secondary analysis restricted to undergraduate educators showed a similar consensus pattern, suggesting that including postgraduate educators did not substantially affect the main findings. The absence of undergraduate students in the expert panel may also be considered a limitation. However, given the objective of operationalising a specialised competency framework and the expectation that learning outcomes should reflect validated clinical and educational expertise, the study prioritised the perspective of experienced clinicians and educators. Future research could usefully explore students’ perspectives regarding the feasibility and perceived relevance of these outcomes in practice. Also, the absence of implementation data limits conclusions regarding educational impact.

The initial item-generation phase involved interpretative synthesis by the research team, which may introduce subjectivity. This was mitigated through the Delphi validation process, which ensured that the outcomes reflected shared expert consensus rather than individual perspectives.

In conclusion, translating a broad competency framework into validated, observable undergraduate learning outcomes provides a practical foundation for more explicit, competency-oriented teaching and assessment in Family Medicine. By supporting learning grounded in real clinical practice, feedback, and progressive responsibility, this approach may inform the future implementation of structured assessment strategies and help prepare physicians to deliver patient-centred care in complex healthcare systems.

## Data Availability

The raw data supporting the conclusions of this article will be made available by the authors, without undue reservation.
