# Supplementary material for: Development and Delphi validation of WONCA-aligned competency-based learning outcomes for undergraduate family medicine internship
Source: Front Med (Lausanne). 2026 Jul 15;13:1899210. doi: 10.3389/fmed.2026.1899210 (PMC13416951; doi:10.3389/fmed.2026.1899210)
Supplement: Supplementary file 1 [file Table_1.docx]

Supplementary Material

**Validated competency-based learning outcomes for the sixth-year Family Medicine internship**

This set of validated competency-based learning outcomes was developed to support teaching, assessment and reflective learning during the sixth-year Family Medicine internship. It aligns with the WONCA competencies for General Practice / Family Medicine speciality and comprises a set of validated learning outcomes that reflect the competencies required in family medicine practice. This framework of validated learning outcomes is not intended to function as a checklist of tasks, nor to provide an exhaustive overview of all clinical situations. Instead, it focuses on meaningful learning experiences that are sufficiently common to occur during the internship and that allow students to demonstrate transversal competencies central to the role of the family physician.

Each learning outcome should be discussed collaboratively between the student and the tutor, using real clinical encounters as the basis for observation, feedback and reflection. Observation may result from one or multiple consultations and should prioritise qualitative feedback over summative judgment.

## Instructions for use

The learning outcomes may be used by tutors and students to support observation, feedback and reflection. Tutors should indicate whether the activity has been observed during clinical practice and provide qualitative feedback. Students should complete the self-reflection section, focusing on the learning gained, the challenges encountered, and the areas for further development. This framework is intended to support ongoing dialogue, feedback and reflective practice throughout the internship.

| **Learning Outcomes** | | **Observed** | **Not Observed** | **Tutor Feedback** |
| --- | --- | --- | --- | --- |
| **Doctor–patient relationship** | 1. In the clinical interview with the patient, the student adopts a person-centred approach to medicine. |  |  |  |
| **Centred on patient and context** | 1. The student analyses personal, family, social and community resources, ensuring that the plan is appropriate to the patient's circumstances. |  |  |  |
| **Promotes patient empowerment** | 1. The student promotes health literacy and checks the patient's understanding of how to manage their problems. |  |  |  |
|  | 1. During the consultation, the student takes a proactive approach, involving the patient in the decision-making process and ensuring that they understand the treatment plan. |  |  |  |
| **Longitudinal Continuity** | 1. The student places the current episode of care seeking within the context of the patient's health history. |  |  |  |
| **Responsible for the health of the community** | 1. The student recognises the health resources available in the community. |  |  |  |
| **Decision-making based on incidence and prevalence** | 1. The student is able to identify the most prevalent health problems in the community and adapt the diagnostic and therapeutic approach to the local context. |  |  |  |
|  | 1. The student orders differential diagnoses based on clinical and epidemiological assessment and the patient's context. |  |  |  |
| **Early undifferentiated stages** | 1. Students analyse clinical cases, developing open and contextualised clinical reasoning capable of integrating diagnostic uncertainty. |  |  |  |
| **Acute and chronic health problems** | 1. The student conducts acute illness consultations and chronic illness monitoring consultations under the direct supervision of the tutor. |  |  |  |
| **Promotes health and well-being** | 1. The student integrates preventive measures and screening appropriate to the individual's context, at different levels of prevention. |  |  |  |
| **Care coordination and advocacy** | 1. The student recognises the role of the family doctor in the healthcare system. |  |  |  |
|  | 1. The student assesses the need for referral to other levels of care from the perspective of integrated care. |  |  |  |
| **First-contact, open access, all health problems** | 1. The student identifies the reasons for seeking medical and non-medical health services. |  |  |  |
|  | 1. The student understands the journey of individuals within the healthcare system. |  |  |  |
| **Physical, psychological, social, cultural and existential dimensions** | 1. Students explore patients' beliefs, values, and psychosocial context, integrating cultural, religious, and social aspects into the assessment and treatment plan. |  |  |  |
| **Clinical Tasks** | 1. The student collects the clinical history in a structured manner, performs a physical examination focused on the patient's problem, and proposes an appropriate care plan under the supervision of the tutor. |  |  |  |
| **Communication with patients** | 1. The student uses appropriate verbal and non-verbal communication tools, promoting empathetic communication. |  |  |  |
| **Management of the practice** | 1. The student prepares a structured record of a consultation period in which they identify the organisational strategies of the clinical activity observed. |  |  |  |
| **Attitude** | 1. The student demonstrates diligence, punctuality and professionalism, reflects on the impact of their beliefs on medical practice and identifies gaps in knowledge to improve their performance. |  |  |  |
| **Science** | 1. The student analyses the risks and benefits of tests and treatments and proposes strategies based on the best available medical evidence. |  |  |  |
| **Context** | 1. The student understands the influence of the organisational context on medical practice. |  |  |  |

Table 1 Competency-based learning outcomes aligned with the WONCA European Definition of General Practice/Family Medicine, for use during the sixth-year Family Medicine internship. For each learning outcome, the tutor indicates whether the activity was observed.

**Student self-reflection section:**
